# Association between dog ownership and mental health among community-dwelling older adults: a cross-sectional study

**DOI:** 10.3389/fpubh.2026.1835505

**Published:** 2026-05-13

**Authors:** Yoko Kuroda, Takafumi Abe, Takehiko Tsujimoto, Masayuki Yamasaki, Minoru Isomura, Atsushi Nagai

**Affiliations:** 1 Department of Neurology, Faculty of Medicine, Shimane University, Izumo, Shimane, Japan; 2 Head Office for Research and Academic Information, Center for Community-based Healthcare Research and Education (CoHRE), Shimane University, Izumo, Shimane, Japan; 3 Faculty of Human Sciences, Shimane University, Matsue, Shimane, Japan

**Keywords:** apathy, community health, depressive symptoms, dog ownership, mental health, older adults

## Abstract

**Introduction:**

Poor mental health in later adulthood poses a significant public health challenge. The association between dog ownership and apathy and depressive symptoms among older adults remains under-researched. We aimed to examine the relationship between dog ownership and apathy and depressive symptoms among community-dwelling older adults.

**Methods:**

This cross-sectional study included 722 community-dwelling adults (356 women) aged ≥65 years. Data on dog ownership were collected using a self-administered questionnaire. Apathy and depressive symptoms were defined as scores ≥16 on the Japanese version of the Starkstein Apathy Scale and ≥40 on the Self-Rating Depression Scale, respectively. Associations were analyzed using logistic regression models to estimate odds ratios (ORs) and 95% confidence intervals (CIs), adjusted for age, sex, body mass index, and multimorbidity.

**Results:**

Dog ownership was reported by 57 participants (7.9%). The high apathy and depressive symptom groups comprised 230 (31.9%) and 224 (31.0%) participants, respectively. Multivariate analysis revealed that dog ownership was associated with a significantly lower prevalence of apathy (OR = 0.49, 95% CI = 0.25, 0.97) compared with dog non-ownership. No association was observed between dog ownership status and depressive symptoms.

**Conclusion:**

Dog ownership was associated with lower odds of apathy among community-dwelling older adults, suggesting a potential role in maintaining motivation in this population.

## 1 Introduction

The global prevalence of mental health issues, such as depression, is approximately 20–35% among older adults (1, 2). This high prevalence constitutes a significant public health concern. Mental health issues among older adults may contribute to the onset of dementia and increased mortality (3, 4). Therefore, it is essential to monitor mental health status at the community level. Among the characteristics of mental health in later life, apathy, manifested as diminished initiative and interest, and depressive symptoms, such as low mood and sadness, are well recognized (5, 6). Apathy and depressive symptoms represent distinct neuropsychiatric syndromes; however, they share overlapping features, making differentiation challenging (7). Nevertheless, accurate identification of each syndrome and linkage to appropriate preventive strategies are important.

Recently, dog ownership has been discussed as a potential means of improving mental health among older adults. For example, dog ownership among socially isolated older adults has been reported to potentially enhance psychological well-being (8). Two review articles have indicated that pet ownership, including dog ownership, is not associated with depressive symptoms but may be beneficial for anxiety (9, 10). However, evidence regarding the impact of pet ownership on mental health among older adults remains limited. A qualitative study reported that dog walking can facilitate social interaction and contribute to psychological well-being (11). An overview of previous research suggests that, although earlier studies have examined the associations between dog ownership and mental health outcomes such as anxiety and depression, very few have specifically investigated its relationship with motivation-related outcomes, particularly apathy. Apathy is a critical psychological condition in older adults that often emerges as an early marker preceding functional decline, contributes to reduced quality of life (12), and is strongly associated with cognitive decline (13). Therefore, although preventing apathy is important, research examining its association with dog ownership remains limited, and this research gap is of considerable importance. Addressing this gap is essential for understanding the potential role of everyday pet ownership as a modifiable factor in promoting mental health among older populations.

Therefore, this study aimed to examine the association between dog ownership and apathy and depressive symptoms among community-dwelling older adults. We hypothesized that dog ownership is associated with a lower prevalence of apathy and depressive symptoms.

## 2 Materials and methods

This cross-sectional study was part of the Shimane CoHRE study conducted by the Center for Community-Based Healthcare Research and Education, Shimane University. Japanese residents with national health insurance are eligible for a health examination in their municipality of residence. Data were collected during a health examination conducted in Unnan city (population: 33,561; proportion aged ≥65 years: 41.38%; area: 553.18 km^2^), Shimane, rural Japan, from August to September 2025. Overall, 724 older adults aged ≥65 years participated in the health examinations. Residents were invited to participate voluntarily in the survey, and no incentives were provided. Inclusion criteria were community-dwelling individuals living independently in their own homes within the municipality who participated in the health examination and consented to respond to the survey. Exclusion criteria included inability to complete the questionnaires. After excluding participants with non-responses to the questionnaire (*n* = 2), data from 722 participants were analyzed. The study protocol was approved by the Medical Research Ethics Committee of the Faculty of Medicine at Shimane University (approval number: 20051214-3), and written informed consent was obtained from all participants before enrollment.

### 2.1 Mental health

Apathy as an emotional function was evaluated using self-administered questionnaires and assessed using the Japanese version of the Starkstein Apathy Scale (14, 15), which comprises 14 items related to motivation and interest, with each item rated on a 4-point Likert scale (0–3). Total scores ranged from 0 to 42, with higher scores indicating a stronger tendency toward apathy. The validity and reliability of the scale have been confirmed (14, 15), and the cut-off point for the Japanese population is 16 (sensitivity, 81.3%; specificity, 85.3%). Participants with a score ≥16 were categorized as having apathy (15). Depressive symptoms were assessed using the Self-Rating Depression Scale (16, 17), designed to evaluate various aspects of depressive states, including concomitant primary emotional, physiological, and psychological symptoms. It comprises 20 items, each rated on a four-point Likert scale. Total scores ranged from 20 to 80, with higher scores indicating stronger depressive symptoms. A cut-off point of 40 was established, and a score ≥40 indicated the presence of depressive symptoms (18).

### 2.2 Dog ownership

Dog ownership was assessed using a self-administered questionnaire. Participants were asked, “Do you own dogs?” with response options of “Yes” or “No”.

### 2.3 Other parameters

Questionnaires were mailed to participants in advance, collected at the health checkup site on the day of examination, and reviewed with participants by trained staff to confirm completeness and clarify any ambiguous responses. In addition to collecting data on basic characteristics (age and sex), body mass index (BMI) was calculated using height and weight measured during the health checkup. Current chronic diseases (hypertension, diabetes mellitus, dyslipidemia, chronic kidney disease, gout, anemia, liver disease, thyroid disorders, cerebrovascular disease, and heart disease) were self-reported and confirmed through interviews conducted by trained staff during the health checkup. Multimorbidity was defined as the presence of two or more chronic diseases (19).

### 2.4 Statistical analysis

Descriptive statistics (frequencies and percentages, as all variables were categorical) were used to compare participant characteristics according to dog ownership status (ownership vs. non-ownership). For comparisons between two groups, the chi-square test was used for categorical variables.

For the primary analysis, multiple logistic regression models were used to examine the associations between dog ownership (reference group: dog non-ownership) and apathy and depressive symptoms, calculating odds ratios (ORs) and 95% confidence intervals (CIs). Model 1 was the crude model. Model 2 was adjusted for age, sex, and BMI. Model 3 was adjusted for multimorbidity in addition to the variables included in Model 2. For all analyses, a two-tailed *p*-value < 0.05 was considered statistically significant. Statistical analyses were performed using IBM SPSS Statistics for Windows, version 29.0.

## 3 Results

Descriptive statistics are presented in Table 1. Dog ownership was reported by 57 participants (7.9%). The high apathy and depressive symptom groups comprised 230 (31.9%) and 224 (31.0%) participants, respectively. When participants were compared according to dog ownership status, significant differences were observed in age (*p* = 0.032) and apathy scores (*p* = 0.034).

**Table 1:** Characteristics of the study participants.

Variables	Total	Dog owner		*p* value
		Ownership	Non-ownership	
	*N* = 722	*n* = 57	*n* = 655	
Sex, *n* (%)				
Men	366 (50.7)	31 (54.4)	335 (50.4)	0.561
Women	356 (49.3)	26 (45.6)	330 (49.6)	
Age, *n* (%)				
65–74 years	231 (32.0)	11 (19.3)	220 (33.1)	0.032
≥75 years	491 (68.0)	46 (80.7)	445 (66.9)	
Body mass index, *n* (%)				
<18.5 kg/m^2^	103 (14.3)	5 (8.8)	98 (14.7)	0.320
18.5–24.9 kg/m^2^	514 (71.2)	41 (71.9)	473 (71.1)	
≥25.0 kg/m^2^	105 (14.5)	11 (19.3)	94 (14.2)	
Multimorbidity, *n* (%)				
≥2 current chronic diseases	263 (36.4)	20 (35.1)	243 (36.5)	0.827
<2 current chronic diseases	459 (63.6)	37 (64.9)	422 (63.5)	
Apathy, *n* (%)				
High (≥16 points)	230 (31.9)	11 (19.3)	219 (32.9)	0.034
Low (<16 points)	492 (68.1)	46 (80.7)	446 (67.1)	
Depressive symptoms, *n* (%)				
High (≥40 points)	224 (31.0)	13 (22.8)	211 (31.7)	0.162
Low (<40 points)	498 (69.0)	44 (77.2)	454 (68.3)	

The *p*-value was derived from the chi-square test. Chi-square values (degrees of freedom): sex, χ^2^ = 0.338 (1); age, χ^2^ = 4.585 (1); body mass index, χ^2^ = 2.276 (2); Multimorbidity, χ^2^ = 0.048 (1); apathy, χ^2^ = 4.496 (1); depressive symptoms, χ^2^ = 1.953 (1).

Table 2 shows the results of the multivariate analysis of the association between dog ownership and mental health. In Model 3, dog ownership was significantly associated with a lower prevalence of apathy compared with dog non-ownership (OR = 0.49; 95% CI = 0.25, 0.97). Consistent results were observed across all models. Depressive symptoms were not associated with dog ownership.

**Table 2:** Associations between dog ownership and mental health among older adults (*n* = 722).

Primary exposure		Apathy			Depressive symptoms		
		OR	(95% CI)	*p* value	OR	(95% CI)	*p* value
Dog ownership (vs. dog non-ownership)	Model 1	0.49	(0.25, 0.96)	0.037	0.64	(0.34, 1.21)	0.165
	Model 2	0.49	(0.25, 0.97)	0.041	0.66	(0.35, 1.26)	0.212
	Model 3	0.49	(0.25, 0.97)	0.039	0.67	(0.35, 1.28)	0.223

Logistic regression analyses were conducted with apathy or depressive symptoms as dependent variables. Model 1 was the crude model. Model 2 included age, sex, and body mass index as covariates. Model 3 was adjusted for multimorbidity in addition to the variables in Model 2. OR, odds ratio; CIs, confidence intervals.

## 4 Discussion

We observed an association between dog ownership and apathy among community-dwelling older adults. Our findings suggest that dog ownership may play a protective role against the decline in motivation among older adults.

The present study found that dog ownership was inversely associated with apathy among community-dwelling older adults, suggesting that dog ownership may play a protective role against the decline in motivation. This finding is supported by prior evidence indicating that pet ownership contributes to psychological well-being in socially isolated older adults in Japan (8), and that dog ownership may improve happiness indices in this population (20). A qualitative study further showed that pet ownership among community-dwelling older adults may foster a sense of companionship, provide purpose and meaning in life, alleviate feelings of loneliness, and promote socialization (21). These psychological benefits may help prevent decreased motivation in social behaviors (apathy) among older adults (22). However, as our study was conducted in a rural area of Japan, the potential cultural and contextual characteristics of this setting should be considered when interpreting the findings. In the study by Taniguchi et al. (23), which focused on older adults living in a rural region of Japan, the combined prevalence of dog and cat ownership was 13.8%. Although direct comparison with our study is not straightforward because we examined dog ownership alone, the overall magnitude appears broadly comparable. Pet ownership prevalence varies considerably across regions and countries (9), which may reflect differences in attitudes toward pet keeping. Future research would benefit from large-scale studies examining regional and cultural variations in pet ownership in greater depth.

The findings of our study showed that dog ownership was not associated with depressive symptoms, similar to findings reported in previous reviews (9, 10). Among older adults in Norway and England (24, 25), dog ownership was not associated with depression. Among older adults in Brazil during the coronavirus disease pandemic, dog ownership was associated with lower levels of depression (26). Conversely, in England, older adults with more depressive symptoms were more likely to own a dog (27); however, pet ownership was not associated with long-term changes in depressive symptoms. Prior research has indicated that increases in apathy and declines in activities of daily living tend to precede mood-related symptoms such as depression and cognitive impairment (13, 28). Depressive symptoms may have a long-term association, as they may be preceded by a decline in motivation. Apathy and depressive symptoms share overlapping features; however, they are underpinned by distinct neurobiological and psychosocial processes (29–32) and can be viewed as separate yet interrelated constructs. One possible explanation for the differential association observed in the present study, an inverse association with apathy but not with depressive symptoms, is that apathy and depression are mediated by partly distinct neural circuits, namely the prefrontal–basal ganglia circuits underlying motivation and the limbic circuits underlying mood regulation. The daily routines, sense of responsibility, and habitual physical activity associated with dog ownership may act preferentially on motivation-related circuits, supporting goal-directed behavior, while having a weaker effect on mood-related symptoms. The present study was conducted among community-dwelling older adults in a rural Japanese setting, a population particularly susceptible to social disengagement and loss of daily purpose due to age-related changes such as retirement, reduced mobility, and bereavement. In this context, the daily routines and sense of responsibility inherent in dog ownership may be especially relevant in maintaining goal-directed motivation, which is the core feature of apathy. This population-specific mechanism may explain why an association was observed with apathy but not with depressive symptoms in our sample. Differences in the association of apathy and depression among dog owners warrant further investigation. From another perspective, dog walking may serve as an opportunity for physical activity (33). Aerobic exercise in older adults may be effective in alleviating mental health stress even at low doses (34). However, further investigation is required to elucidate the mechanisms underlying the relationship between dog ownership and mental health. Of the baseline characteristics, age differed significantly between dog owners and non-owners (*p* = 0.032), with dog owners more frequently aged ≥75 years, whereas BMI did not differ significantly (*p* = 0.320). The age difference may reflect cohort-specific patterns of pet keeping in this rural area, in which long-term ownership of a household dog is more common among older residents. To minimize confounding, age and BMI were adjusted for in Models 2 and 3; the inverse association with apathy persisted across these analyses, suggesting that the observed association is unlikely to be explained by these baseline differences alone.

From a public health perspective, our findings have several implications. Apathy is increasingly recognized as a modifiable target for the prevention of cognitive and functional decline in older adults, yet community-based interventions specifically targeting prevention of motivational decline remain scarce. The observed association between dog ownership and lower odds of apathy suggests that everyday human–animal interaction may represent one potential avenue for sustaining motivation in later life. In rural settings such as the present study area, where access to organized social activities can be limited, pet-related programs (e.g., community dog-walking groups, supported pet care for older residents) may offer a feasible avenue for promoting mental health. Furthermore, given that apathy often emerges before clinically apparent depression or cognitive impairment, our results support the value of including apathy assessments in routine community health checkups for early identification of at-risk individuals.

This study had some limitations. First, due to its cross-sectional design, causal relationships cannot be established; it remains possible that higher motivation leads to dog ownership rather than vice versa. Second, given the single rural municipality setting and inclusion of health-check attendees, generalizability to non-attendees or urban populations may be limited. Third, participation rates could not be ascertained because of the fragmented nature of health checkup systems in Japan; therefore, the representativeness of the sample relative to the broader older adult population remains uncertain, and selection bias cannot be excluded. Fourth, the number of dog owners was limited to 57. A sensitivity *post hoc* power analysis (total sample size = 722, outcome prevalence = 33%, two-sided *α* = 0.05, power = 80%, assuming a binomial distribution of the exposure and R^2^ = 0.2 for other covariates) indicated a minimum detectable ORs of approximately 0.63, suggesting adequate power to detect moderate associations but potential underpowering for very small effect sizes. Fifth, as the dog ownership questionnaire consisted of a single yes/no item, it could not capture qualitatively important aspects such as duration of ownership, responsibility for daily care, indoor versus outdoor keeping, or the strength of the human–animal bond. This simplicity may have led to misclassification (for example, participants who shared caregiving responsibilities for a dog owned by another household member, making it unclear who should be considered the primary caregiver) and, as the direction of this misclassification is uncertain, may have resulted in either overestimation or underestimation of the associations. Finally, several potential confounders were not measured, including socioeconomic status, ownership of other pets, housing arrangement, exercise habits, functional status, and social isolation, which could influence dog ownership and apathy. Considering these limitations, future studies using longitudinal designs and more rigorous assessments are warranted.

In conclusion, our study showed that dog ownership was associated with a lower prevalence of apathy among community-dwelling older adults. Our findings suggest that dog ownership may be associated with maintained motivation, although causal inference is precluded by the cross-sectional design. While pet ownership offers mental health benefits for older adults, it also presents potential challenges, such as financial costs and risks of allergies, asthma, or zoonotic infections. Furthermore, the loss of a pet can impose a significant emotional and psychological burden on this population (35). Therefore, further discussion is warranted from various public health perspectives.

## Data Availability

Data are available upon reasonable request but not publicly available because of privacy/ethical restrictions.
